# VGLUTs and Glutamate Synthesis—Focus on DRG Neurons and Pain

**DOI:** 10.3390/biom5043416

**Published:** 2015-12-02

**Authors:** Mariana Malet, Pablo R. Brumovsky

**Affiliations:** Institute of Research on Translational Medicine, Consejo Nacional de Investigaciones Científicas y Técnicas (CONICET)—Austral University, Avenida Juan D. Perón 1500, Pilar, Buenos Aires 1629AHJ, Argentina; E-Mail: mariana_malet@hotmail.com

**Keywords:** axotomy, DRG, glutamate, neuropathy, neuropeptides, pain, peripheral nerves, sensory neurons, vesicular glutamate transporter, visceral organs

## Abstract

The amino acid glutamate is the principal excitatory transmitter in the nervous system, including in sensory neurons that convey pain sensation from the periphery to the brain. It is now well established that a family of membrane proteins, termed vesicular glutamate transporters (VGLUTs), serve a critical function in these neurons: they incorporate glutamate into synaptic vesicles. VGLUTs have a central role both under normal neurotransmission and pathological conditions, such as neuropathic or inflammatory pain. In the present short review, we will address VGLUTs in the context of primary afferent neurons. We will focus on the role of VGLUTs in pain triggered by noxious stimuli, peripheral nerve injury, and tissue inflammation, as mostly explored in transgenic mice. The possible interplay between glutamate biosynthesis and VGLUT-dependent packaging in synaptic vesicles, and its potential impact in various pain states will be presented.

## 1. Vesicular Glutamate Transporters in Primary Afferent Neurons

**Figure 1:**
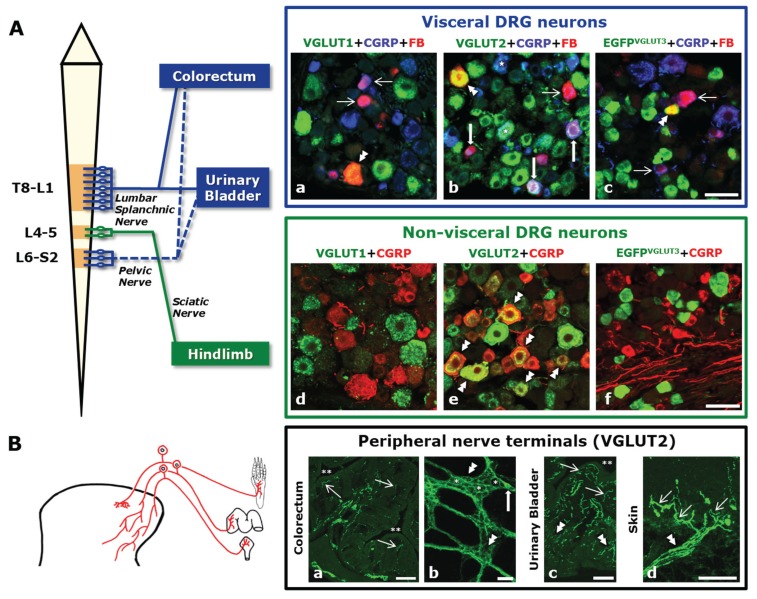
(**A**) (Left panel) Schematic diagram showing the spinal cord and the dorsal root ganglia (DRG) levels involved in the formation of the lumbar splanchnic, pelvic, and sciatic nerves. Colorectum and urinary bladder are examples of visceral pelvic organs receiving dual innervation by the lumbar splanchnic and pelvic nerves. Note that, for clarity, only L4–5 DRGs are depicted as supplying the sciatic nerve and innervating non-visceral tissues in the hindlimb (even though a small contribution of L6 is also common). (Top right panel) Fluorescence micrographs of DRG sections incubated with calcitonin gene-related peptide (CGRP) and vesicular glutamate transporter-1 (VGLUT1), VGLUT2, or EGFP antisera (EGFP is used as a reporter gene of VGLUT3) and where labeled urinary bladder-projecting neurons can be detected by their content of the retrograde tracer fast blue (previously injected into the subserosal space). Some urinary bladder neurons express VGLUT1 or EGFP^VGLUT3^ (double arrowheads in a, c); many others only express calcitonin gene-related peptide (CGRP) (thin arrows in a, c). In contrast, VGLUT2 is commonly expressed by urinary bladder neurons (double arrowhead and thick arrows in b), often along with CGRP (thick arrows in b). Bottom right panel: Fluorescence micrographs of L4-5 DRG sections incubated with CGRP and VGLUT1, VGLUT2, or EGFP antisera. Many neurons express VGLUT2, in most cases coexpressing with CGRP (double arrowheads in e). VGLUT1 and VGLUT3 appear more modestly expressed, and virtually never in association with CGRP (d, f). Many VGLUT1-, VGLUT2- and EGFP^VGLUT3^-only visceral and non-visceral DRG neurons can also be seen (d–f). (**B**) Left panel: Schematic diagram showing three representative DRG neurons projecting towards visceral organs (urinary bladder and colorectum) or the hindpaw (the central projections of these neurons and their termination (widespread distribution for visceral endings; narrow distribution for cutaneous endings) in the dorsal horn are also depicted). Right panel: (double asterisk indicates visceral organ lumen) The profuse VGLUT2 expression in nerve terminals reaching the colorectum (a, b), the urinary bladder (c) and the hindpaw glabrous skin (d) is shown. Many nerve terminals in the colorectal mucosa (thin arrows in (a) as well as myenteric plexus (double arrowheads in b) express VGLUT2. Within the myenteric plexus islets, several neurons lacking VGLUT2 can be observed (single asterisks in b), although occasional neurons expressing the transporter can be detected (thick arrow in b). In the urinary bladder, a profuse VGLUT2-expressing neuropil is detected both in muscular (double arrowheads in c) and mucosal layers (thin arrows in c). Finally, the skin is also provided with abundant nerve terminals expressing VGLUT2 (thin arrows in d) stemming from thick nerve bundles (double arrowhead in d). Scale bars: 50 µm (A: a–f; B: b); 100 µm (B: d); 200 µm (B: a, c). (Immunohistochemical techniques used in A: a–f and B: a–c are the same as in [7,8,9]; Figure B, subfigure d has been partially modified (with permission) from [9]).

Primary afferent neurons, typically populating the dorsal root ganglia (DRG), are the first relay neurons transmitting both innocuous and painful stimuli (mechanical, thermal and chemical) towards the spinal cord. DRG neurons can be further subdivided into: (1) non-visceral, localized, for example, in the lumbar 4–5 DRGs and transmitting afferent input from muscle and skin of the leg by way of the sciatic nerve [1]; and (2) visceral, such as those located at the thoracolumbar (Thoracic 8-Lumbar 1 DRGs) and lumbosacral (Lumbar 6-Sacral 2) DRGs, transmitting afferent input from visceral organs like the colorectum and the urinary bladder by way of the lumbar splanchnic (thoracolumbar) and pelvic (lumbosacral) nerves (Figure 1A) [2]. DRG neurons at these different levels send out axons that terminate in visceral and non-visceral tissues (Figure 1B), in many cases as free nerve endings called nociceptors, a high-threshold sensory receptor capable of transducing and encoding painful stimuli (International Association for the Study of Pain (IASP); http://www.iasp-pain.org).

Glutamate is the most abundant excitatory neurotransmitter in DRG neurons and is therefore positioned as a strategic player in the transmission of innocuous stimuli, as well as of nociceptive or neuropathic pain (see Box 1 for terminology). The former is defined as a painful signal occurring in a normally functioning somatosensory nervous system, during actual or threatened damage to non-neural tissue, and due to activation of nociceptors. The latter arises from nerve or tissue damage causing pain for prolonged periods of time (IASP).

Box 1 **Brief description of specific terminology and pain-like behavior tests mentioned throughout this review.**

**(1)**  **Terminology ^(IASP)^**
  Hyperalgesia: Increased pain from a stimulus that normally provokes pain.
  Allodynia: Pain due to a stimulus that does not normally provoke pain.
  Neuropathic pain: Pain caused by a lesion or disease of the somatosensory nervous system.
  Nociceptive pain: Pain that arises from actual or threatened damage to non-neural tissue and is due to the activation of nociceptors.
**(2)**  **Pain-Like Behavior Tests**
  (A) Thermal pain threshold *
    a) Tail withdrawal (heat sensitivity) test [3]: The distal half of the tail is dipped into a thermostatically controlled warm-hot water bath.
    b) Hot plate test [3]: Animal is placed on a warmed metal plate.
    c) Hargreaves test [3]: The plantar surface of the hindpaws is exposed to radiant heat, producing a continuous but discrete rise in temperature.
    d) Acetone test [3]: The plantar surface of the hindpaws is exposed to a droplet of acetone.
    e) Cold ethanol test [4]: The distal half of the tail is immersed in −14 °C to −15 °C ethanol.
    f) Dry ice pellet test [5]: The plantar surface of the hindpaws is exposed to a dry ice pellet 1 cm in diameter.
  (B) Mechanical pain threshold * [3]
    a) Randall-Sellito test: The plantar or dorsal surfaces of the hindpaws are exposed to increasing mechanical force (tip of the device). The maximum force applied is limited to 250 g to avoid skin damage.
    b) Von Frey test: The plantar surface of the hindpaw is exposed to a series of nylon monofilaments of different thicknesses, exerting varying degrees of pressure. The lesser monofilament inducing hindpaw withdrawal is recorded.
  (C) Itch
    a) Spontaneous itch behavior [6]: Itch behavior is recorded for 30 min using a digital video camera. One bout of scratching by either hindpaw is defined as a scratching episode and quantified.

* Response latency and/or number of events (withdrawal of tail or hindpaw, flickering, licking, lifting) are used as measurable outcome.

The synthetic machinery involved in glutamate production is present in DRG neurons, including, in order of importance: (1) the critical mitochondrial enzyme glutaminase (GLS), which converts glutamine into glutamate; (2) the tricarboxylic acid cycle for the conversion of glucose into glutamate; and (3) the *N*-acetylated α-linked acidic dipeptidase, participating in the suggested degradation of *N*-acetyl-aspartyl-glutamate into glutamate and *N*-acetyl-aspartate (see [10] for more details). In fact, GLS is expressed by the majority of DRG neurons [11], although small and medium-sized DRG neurons seem to exhibit the highest amounts as evaluated with immunohistochemistry [11,12]; importantly, most DRG neurons expressing GLS also synthesize the neuropeptidergic marker calcitonin gene-related peptide (CGRP) [12]. Finally, the enzymatic activity of GLS has been detected not only in DRGs, but also in dorsal roots and the sciatic and trigeminal nerves, suggesting local (axons, nerve endings) glutamate biosynthesis [10].

**Figure 2:**
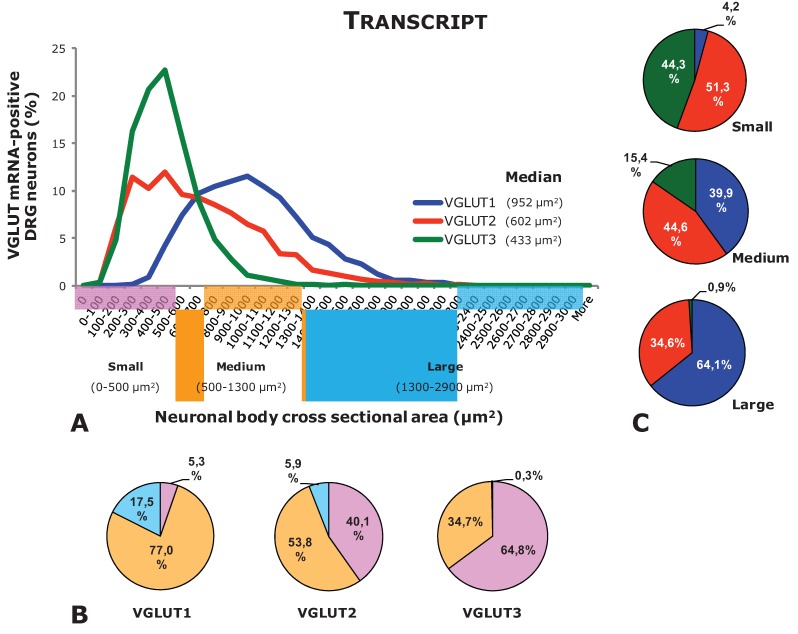
Neuronal size distribution of DRG neurons positive for VGLUT1-VGLUT3 mRNA (based on data from [13]). (**A**) Histogram showing the size distribution of L4-5 DRG neurons expressing the transcript of VGLUT1, VGLUT2, or VGLUT3 (Percentages were obtained comparing cell soma size at each value in “X” with the total number of neurons measured for each VGLUT). (**B**) Pie charts showing the percentage of small, medium-sized, and large DRG neurons relative to each VGLUT. (**C**) Pie charts showing the percentage of VGLUT1-, VGLUT2-, or VGLUT3-positive DRG neurons relative to each neuronal size.

**Figure 3:**
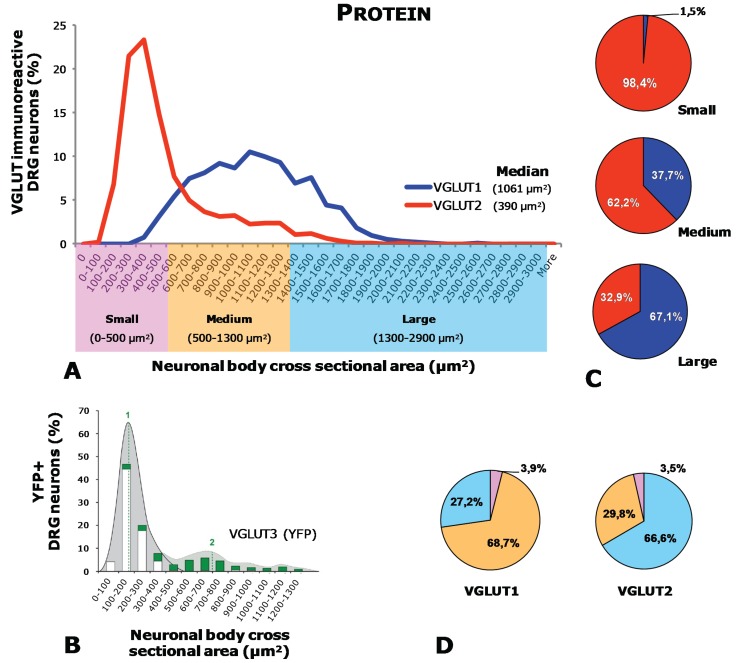
Neuronal size distribution of DRG neurons positive for VGLUT1-VGLUT3 protein (based on data from [9]). Figure in B was taken from [31] and partially modified with kind permission of Prof. Sandkühler). (**A**) Histogram showing the size distribution of L4–5 DRG neurons immunoreactive for VGLUT1 or VGLUT2 (Percentages were obtained comparing cell soma size at each value in “X” with the total number of neurons measured for each VGLUT)*.* (**B**) Histogram showing the size distribution of Yellow Fluorescent Protein-positive (used here for identification of VGLUT3) DRG neurons. (**C**) Pie charts showing the percentage of small, medium-sized, and large DRG neurons relative to VGLUT1 or VGLUT2. (**D**) Pie charts showing the percentage of VGLUT1- or VGLUT2-positive DRG neurons relative to each neuronal size.

Once synthesized, glutamate in primary afferent neurons is incorporated into synaptic vesicles by means of vesicular glutamate transporters (VGLUTs) located in the membrane of synaptic vesicles. So far three types of VGLUTs have been identified: VGLUT1-3 (for details on structure, sequence homologies, functionality, and localization of VGLUTs, we recommend reading references [14,15,16,17,18,19,20,21,22,23,24]). Extensive analysis, mostly done in mice, has shown that these three transporters are to a certain extent expressed in different subpopulations of DRG neurons. Thus, VGLUT2 is the most broadly expressed transporter, found in a large number of visceral [7,8] and non-visceral [6,9,25,26,27] peptidergic and/or non-peptidergic [6,7,8,9,25,27,28] DRG neurons (Figure 1A, Table 1) of different sizes (large, small, and medium-sized) (Figure 1A, Figure 2 and Figure 3). In contrast, VGLUT1 and VGLUT3 are observed in subpopulations of mostly large and medium-sized (VGLUT1) or small (VGLUT3) visceral [7,8,29] and non-visceral [9,29,30,31,32,33] DRG neurons (Figure 1A, Figure 2 and Figure 3; Table 1). Moreover, most VGLUT1 or VGLUT3 DRG neurons are non-peptidergic and IB4-negative [7,8,9,29,30,31]. Synthesized VGLUT2 in DRG neurons undergoes an important peripheral axonal transport, as shown by the abundance of VGLUT2-expressing nerve terminals found in viscera [7,8] as well as the hindpaw skin [9] (Figure 1B). In contrast, VGLUT1 is observed in a small number of nerve terminals in the hindpaw skin, whereas VGLUT3 appears to most specifically innervate hairy skin [33]. However, segregation between the different VGLUTs, at least as observed within the cell bodies of DRG neurons, is not complete. This is most clear in the subpopulation of tyrosine hydroxylase (TH)-expressing, non-visceral DRG neurons [34], all of which co-express both VGLUT2 [29,35] and VGLUT3 [29,31,33,35,36,37]. A certain degree of coexpression possibly also occurs between VGLUT1 and VGLUT2, as judged by the overwhelming wide expression of the latter in the great majority of DRG neurons [7,8,9,25] and supported in recent studies using transgenic mice [38]. In the rat, VGLUTs expression in DRG neurons has been studied to a lesser extent, although presence of VGLUT1 transcript and protein in large and medium-sized DRG neurons has been described [39].

**Table 1:** Percentages of visceral and non-visceral DRG neurons expressing transcript and protein of VGLUT1-VGLUT3. Dashes shown data that has not been assessed.

Target	DRG level	Vglut1		Vglut2		Vglut3	
		Protein	Transcript	Protein	Transcript	Protein	Transcript
Hindpaw	L4-5	∼37% [32]	∼40% [13]	∼65% [9]	∼70% [13]	∼10% [30]	∼17% [13]
		∼12% [9]		∼90% [25]	∼80% [25]	∼19% [33]	∼16% [33]
Colorectum	T8-L1	∼15% [8]	—	∼98% [8]	—	∼17% [29]	—
	L6-S2	∼8% [8]	—	∼97% [8]	—	∼4% [29]	—
Urinary bladder	T8-L1	∼39% [7]	—	∼94% [7]	—	∼28% [7]	—
	L6-S2	∼26% [7]	—	∼94% [7]	—	∼8% [7]	—

In conclusion, the abundant expression of VGLUT2 in DRG neurons, plus its active central and peripheral axonal transport [7,8,9,29], suggests a relevant role in the incorporation of glutamate by synaptic vesicles for their action at nerve endings. For VGLUT1 and VGLUT3, this may also be true for those specific neuronal subpopulations where they were expressed, and eventually also sharing roles with VGLUT2. In the next section, the relative importance of each VGLUT to different pain modalities will be described based on extensive studies in transgenic mice.

## 2. Role of VGLUTs in Pain Mechanisms—Lessons from Transgenic Mice

### 2.1. Global Deletion of VGLUTs

Most of the current knowledge on the role of VGLUTs in pain mechanisms (frequently in relation to non-visceral pain) has been gathered by studying global (DRGs, spinal cord, brainstem, and brain) or conditional (targeting specific subpopulations of DRG neurons) knock-out (KO) mice (Table 2, Table 3 and Table 4) (see Box 1 and Box 2 for pain models and behavioral pain tests described in this review). In particular, for global KO strategies, homozygote VGLUT1 or VGLUT2 deletion results in perinatal lethality [40,41] (not so for homozygote VGLUT3-KO mice [30,31]). However, heterozygote VGLUT1- or VGLUT2-KO mice were designed and successfully used to evaluate the consequences on rodent behavior and vesicle filling in adult life [40,41]. The following sections summarize the key observations made through these different KO strategies.

Box 2 **Brief description of inflammatory and neuropathic pain models mentioned throughout this review.**

(1) Inflammatory Pain Models [42] ^+^
  a) Nerve growth factor (NGF)
  b) Capsaicin
  c) Formalin
  d) Carrageenan
  e) CFA (complete Freund´s adjuvant)
(2) Neuropathic Pain Models [1] ^*^
  a) Spared nerve injury (SNI): Tibial and common peroneal nerves (two of the three branches of the sciatic nerve) are axotomized (completely sectioned), while the sural nerve is left intact.
  b) Chronic constriction injury (CCI): Three to four light ligatures are placed around the sciatic nerve at the mid thigh level.
  c) Partial sciatic nerve injury (PSNL): A tight ligation of the dorsal third to half of the common sciatic nerve at the upper-thigh level is produced.
  d) Incisional pain: Skin of the plantar surface of the hindpaw is cut using a sharp blade, followed by suturing.
  e) Oxaliplatin (antineoplastic drug)-induced pain: Oxaliplatin is administered systemically ^#^.

^+^ These different models consist of an intraplantar injection of various noxious chemical irritants/inflammogens that result in variable degrees of allodynia and hyperalgesia. Following the initial injection, pain can be measured minutes to days later, at the site of inflammation or away from the primary site of injury using the tests described in Box 1.

* These different models result in variable degrees of allodynia and hyperalgesia manifested by decreased thermal and mechanical response thresholds. These are measured using the tests described in Box 1.

# The oxaliplatin model evokes mechanical allodynia and hyperalgesia, as well as cold allodynia, with delayed onset, gradually increasing severity, a distinct delay to peak severity, and duration of about 2.5 months. There is no effect on heat sensitivity.

#### 2.1.1. Global Deletion of VGLUTs and Effect on Nociceptive or Inflammatory Pain

The effects on nociceptive pain transmission are rather modest in global heterozygote VGLUT1- or VGLUT2-KO mice [40,41] or homozygote VGLUT3-KO mice [30,31] (Table 2). The effect of VGLUT1 or VGLUT2 deletion on inflammatory pain is also negligible [40,41], whereas VGLUT3-KO mice do show reduced capsaicin- or carrageenan-induced mechanical hypersensitivity [30,31] (Table 3). VGLUT3 may also have a role in carrageenan-induced thermal hypersensitivity, although this is currently under debate, since both reduction [30] or no change [31] have been reported. However, data on conditional VGLUT3-KO mice seems to refute a role in thermal hypersensitivity (see below).

**Table 2:** Effects on nociceptive pain and itch of various types of VGLUT deletion (Reductions are shown in red, lack of change are shown in black and dashes show pain modalities that have not been assessed; concomitant actions with neuropeptides are shown in purple; # Brumovsky, unpublished data; * Yes, when concomitant pharmacological blockade of SP).

Nociceptive Pain							Itch
	Thermal			Mechanical		Visceral	
Tail Withdrawal	Hot Plate	Hargreaves	Acetone, Cold ethanol, Dry ice pellet	Randall-Sellito	Von Frey		
**Global KO**							
**VG1 (+/−)** [40]	**VG1 (+/−)** [40]	**—**	**—**	**—**	**VG1 (+/−)** [40]	**VG1 (+/−)** [40]	—
**VG2 (+/−)** [40,41]	**VG2 (+/−)** [40,41]	**VG2 (+/−)** [40,41]	**—**	**—**	**VG2 (+/−)** [40]	**VG2 (+/−)** [40]	—
**—**	**VG3 (−/−)** [30,31]	**VG3 (−/−)** [30]	**VG3 (−/−)** [30,31]	**VG3 (−/−)** [30]	**VG3 (−/−)** [30,31]	**VG3 (−/−)** ^#^	—
**Targeted KO**							
**VG2-DRG** [26]	**—**	**VG2-DRG** [26]	—	**VG2-DRG** [26]	**VG2-DRG** [26]	**—**	**VG2-DRG** [26]
**VG2-nocicept.** [25]	**VG2-nocicept.** [25]	**VG2-nocicept.** [25]	**VG2-nocicept.** [25]	**VG2-nocicept.** [25]	**VG2-nocicept.** [25]	**—**	**VG2-nocicept.** [25]
**—**	**—**	**VG2-TRPV1** [6,28,38]	**VG2-TRPV1** * [38]	**VG2-TRPV1** [6]	**VG2-TRPV1** [6,28,38]	**—**	**VG2-TRPV1** [6,28,38]
**—**	**VG2- TH** [6]	**VG2 –TH** [6]	**—**	**VG2 –TH** [6]	**VG2 –TH** [6]	**—**	**VG2-TH** [6]
**—**	**VG2-Nav1.8** [27]	**VG2-Nav1.8** [6,27]	**VG2-Nav1.8** [27]	**VG2-Nav1.8** [27]	**VG2- Nav1.8** [27]	**—**	**VG2-Nav1.8** [6,27]
**—**	**—**	**VG3-DRG** [33]	**—**	**VG3-DRG** [33]	**VG3- DRG** [33]	**—**	—

VG1 (+/−), VGLUT1-KO; VG2 (+/−), VGLUT2-KO; VG3 (−/−), VGLUT3-KO; VG2-DRG, VGLUT2-DRG-KO; VG2-nocicept., VGLUT2-nociceptors-KO; VG2-TRPV1, VGLUT2-TRPV1-KO; VG2-TH, VGLUT2-TH-KO; VG2-Nav1.8, VGLUT2-Nav1.8-KO; VG3-DRG, VGLUT3-DRG-KO.

**Table 3:** Effects on nociceptive pain and itch of various types of VGLUT deletion (Reductions are shown in red, lack of change are shown in black and dashes show pain modalities that have not been assessed; concomitant actions with neuropeptides are shown in purple; & No hyperalgesia according to Draxler *et al*. 2014 [31]; δ Yes, when concomitant pharmacological blockade of CGRP and/or SP; ^+^ Yes, when concomitant pharmacological blockade of SP and CGRP; £ Yes, when concomitant pharmacological blockade of CGRP; * Yes, when concomitant pharmacological blockade of SP).

Inflammatory Pain										
NGF		Capsaicin		Formalin		Carrageenan			CFA	
Mech.	Heat	Mech.	Licking Biting	Phase I	Phase II	Mech.	Heat	Cold	Mech.	Heat
**Global KO**										
**—**	**—**	**—**	**—**	**VG1 (+/−)** [40]	**VG1 (+/−)** [40]	**—**	**VG1 (+/−)** [40]	**—**	**—**	**—**
**—**	**—**	**—**	**—**	**VG2 (+/−)** [40,41]	**VG2 (+/−)** [40,41]	**—**	**VG2 (+/−)** [40,41]	**—**	**—**	**—**
**—**	**—**	**VG3 (−/−)** [30]	**—**	**VG3 (−/−)** [30]	**VG3 (−/−)** [30]	**VG3 (−/−)** [30,31]	**VG3 (−/−) ^&^** [30,31]	**VG3 (−/−)** [31]	**—**	**—**
**Targeted KO**										
**VG2-DRG** [26]	**VG2-DRG** [26]	**—**	**—**	**VG2-DRG** [26]	**VG2-DRG** [26]	**VG2-DRG** [26]	**VG2-DRG** [26]	**—**	**—**	**—**
**—**	**—**	**—**	**VG2-nocicept.** [25]	**VG2-nocicept.** [25]	**VG2-nocicept.** [25]	**VG2-nocicept.** [25]	**VG2-nocicept.** [25]	**—**	**VG2-nocicept.** [25]	**VG2-nocicept.** [25]
**VG2-TRPV1 ^δ^** [28]	**VG2-TRPV1 ^+^** [28]	**—**	**—**	**VG2-TRPV1 ^£^** [6,28,38]	**VG2-TRPV1 ^+^** [6,28,38]	**VG2-TRPV1 ^+^** [28]	**VG2-TRPV1 ^+^** [28]	**—**	**—**	**—**
**—**	**—**	**—**	**—**	**VG2-TH** [6]	**VG2-TH** [6]	**—**	**—**	**—**	**—**	**—**
**—**	**VG2- Nav1.8** [27]	**—**	**—**	VG2- Nav1.8* [27]	VG2- Nav1.8 * [27]	**—**	**—**	**—**	**—**	**—**
**—**	**—**	**—**	**VG3-DRG** [33]	**—**	**—**	**VG3-DRG** [33]	**VG3-DRG** [33]	**—**	**VG3-DRG** [33]	**VG3-DRG** [33]

VG1 (+/−), VGLUT1-KO; VG2 (+/−), VGLUT2-KO; VG3 (−/−), VGLUT3-KO; VG2-DRG, VGLUT2-DRG-KO; VG2-nocicept., VGLUT2-nociceptors-KO; VG2-TRPV1, VGLUT2-TRPV1-KO; VG2-TH, VGLUT2-TH-KO; VG2-Nav1.8, VGLUT2-Nav1.8-KO; VG3-DRG, VGLUT3-DRG-KO.

**Table 4:** Effects on nociceptive pain and itch of various types of VGLUT deletion (Reductions are shown in red, lack of change are shown in black and dashes show pain modalities that have not been assessed).

Neuropathic Pain												
SNI		CCI			PSNL			Incisional		Oxaliplatin		
Mech.	Cold	Mech.	Hargr.	Cold	Mech.	Hargr.	Cold	Mech.	Hargr.	Mech.	Hargr.	Cold
**Global KO**												
**VG1 (+/−)** [40]	**VG1 (+/−)** [40]	**VG1 (+/−)** [40]	**—**	**VG1 (+/−)** [40]	**—**	**—**	**—**	**—**	**—**	**—**	**—**	**—**
**VG2 (+/−)** [40,41]	**VG2 (+/−)** [40,41]	**VG2 (+/−)** [40]	**—**	**VG2 (+/−)** [40]	**—**	**—**	**—**	**—**	**—**	**—**	**—**	**—**
**VG3 (−/−)** [30]	**—**	**VG3 (−/−)** [31]	**VG3 (−/−)** [31]	**VG3 (−/−)** [31]	**—**	**—**	**—**	**VG3 (−/−)** [30]	**VG3 (−/−)** [30]	**VG3 (−/−)** [31]	**VG3 (−/−)** [31]	**VG3 (−/−)** [31]
**Targeted KO**												
**—**	**—**	**—**	**—**	**—**	**VG2-DRG** [26]	**VG2-DRG** [26]	**VG2-DRG** [26]	**—**	**—**	**—**	**—**	**—**
**—**	**—**	**VG2-nocicept.** [25]	**VG2-nocicept.** [25]	**—**	**—**	**—**	**—**	**—**	**—**	**—**	**—**	**—**
**—**	**—**	**—**	**—**	**—**	**VG2-TRPV1** [43]	**VG2-TRPV1** [43]	**VG2-TRPV1** [43]	**—**	**—**	**—**	**—**	**—**
**—**	**—**	**—**	**—**	**—**	**VG2-Nav1.8** [27]	**VG2-Nav1.8** [27]	**VG2-Nav1.8** [27]	**—**	**—**	**—**	**—**	**—**
**VG3-DRG** [33]	**—**	**—**	**—**	**—**	**—**	**—**	**—**	**—**	**—**	**—**	**—**	**—**

VG1 (+/−), VGLUT1-KO; VG2 (+/−), VGLUT2-KO; VG3 (−/−), VGLUT3-KO; VG2-DRG, VGLUT2-DRG-KO; VG2-nocicept., VGLUT2-nociceptors-KO; VG2-TRPV1, VGLUT2-TRPV1-KO; VG2-TH, VGLUT2-TH-KO; VG2-Nav1.8, VGLUT2-Nav1.8-KO; VG3-DRG, VGLUT3-DRG-KO.

#### 2.1.2. Global Deletion of VGLUTs and Effect on Neuropathic Pain

When analyzing the impact of global VGLUT deletion on peripheral nerve injury, roles for VGLUT2 and VGLUT3 are exposed. Thus, the mechanical hypersensitivity resulting from the spared nerve injury (SNI) model is reduced in global heterozygote VGLUT2-KO [40,41] or homozygote VGLUT3-KO [30] mice. Also, in a model of chemotherapy-induced neuropathic pain using the antineoplastic drug cyclic oxyplatin [31], or after hindpaw skin incision [30], mice with deleted VGLUT3 exhibit decreased mechanical [30,31] and cold hypersensitivity [31], compared to WT mice. In contrast, excluding the observation of reduced cold hypersensitivity in VGLUT2-KO mice [40,41], global deletion of VGLUT1, VGLUT2 [40,41], or VGLUT3 [31] does not affect the processing of mechanical and thermal pain during chronic constriction injury (CCI) (Table 4).

While remaining a useful tool, a caveat of VGLUT global deletion is the effect at multiple sites, and therefore the relative contribution at each neuronal location is poorly addressed. Moreover, partial global deletions (heterozygote VGLUT1- and VGLUT2-KO mice) to avoid premature death can also hinder interpretations, since only about 50% of the content for a given deleted VGLUT is lost [37]. In order to circumvent such limitations, and thanks to technological improvement, a number of new VGLUT-KO mice have begun to emerge, allowing for selective targeting at the primary afferent level. Such an approach has identified in more detail the contribution of mostly VGLUT2 and VGLUT3 in specific pain modalities, as well as exposing interesting interactions between glutamatergic and peptidergic neurotransmitters.

### 2.2. Conditional Deletion of VGLUTs

#### 2.2.1. VGLUT2-DRG-KO Mice

In contrast to the virtual failure of global VGLUT2 deletion in affecting the transmission of nociceptive pain, its selective deletion in all DRG neurons expressing it (called here VGLUT2-DRG-KO mice) showed instead its essential role in pain mechanisms. Impairment of virtually all pain modalities, including nociceptive thermal (heat and cold) and mechanical sensations (Table 2), is observed in these mice. Such a crucial role also extends to pain responses to formalin, NGF-induced, and carrageenan-induced thermal and mechanical hyperalgesia [26] (Table 3). Furthermore, the importance of VGLUT2 exposed in global VGLUT2-KO mice during neuropathic pain was confirmed in VGLUT2-DRG-KO mice, since they also manifest a reduction in mechanical allodynia and an absence of cold allodynia and heat hyperalgesia after partial sciatic nerve ligation (PSNL) [26] (Table 4).

#### 2.2.2. VGLUT2-Nociceptors-KO Mice

An even more restrictive deletion of VGLUT2 in DRG neurons, specifically in nociceptors (peptidergic and non-peptidergic C and Aδ neurons; called here VGLUT2-nociceptors-KO mice) [25] also results in decreased responses to nociceptive heat (but not cold), mechanical, and chemical (capsaicin) stimuli (Table 2 and Table 3), as well as complete abolishment of heat hypersensitivity during intraplantar carrageenan or complete Freund’s adjuvant (CFA) (Table 3), or after CCI (Table 3). However, and in contrast to VGLUT2-DRG-KO mice, VGLUT2-nociceptors-KO mice maintain normal responses to intraplantar formalin (Table 3), as well as unaffected mechanical hypersensitivity during intraplantar carrageenan or CFA (Table 3), or after CCI (Table 4) [25].

#### 2.2.3. VGLUT2-TRPV1-KO Mice

The participation in thermal hyperalgesia was further analyzed in mice with deleted VGLUT2 in DRG neurons expressing heat, pH, and capsaicin sensing transient receptor potential cation channel, subfamily V, member 1 (TRPV1) (called here VGLUT2-TRPV1-KO mice) [6,38,43]. As expected, these mice exhibit reduced sensitivity to nociceptive heat (Table 2). However, further analysis exposed an apparently crucial interaction between the glutamatergic and peptidergic signaling systems in the transmission of various pain modalities by this subset of TRPV1-expressing DRG neurons. Thus, systemic blockade of CGRP further decreases nociceptive heat in these mice [6,28]. Moreover, VGLUT2-TRPV1-KO mice exhibit reduced hypersensitivity to noxious cold (tail immersion in −15 °C acetone) only with concomitant inhibition of substance P (but not CGRP) [38] (Table 2). Such an interaction also appears during inflammatory pain, since control and transgenic mice do not differ in their heat or mechanical hyperalgesia after intraplantar NGF or carrageenan, or their painful responses to the intraplantar injection of formalin, unless SP and/or CGRP signaling is systemically blocked in the transgenic mice [28] (Table 3). Altogether, nociceptive heat and cold sensations, as well as heat and mechanical hypersensitivities during peripheral tissue inflammation transmitted by TRPV1-expressing DRG neurons appear to be dependent, to a greater or lesser extent, on the interaction of both glutamatergic and peptidergic signaling [6,28,38]. Finally, VGLUT2-TRPV1-KO mice show a dramatic reduction in heat (but not cold) and mechanical hyperalgesia after PSNL, compared with WT mice [43] (Table 4).

#### 2.2.4. VGLUT2-TH-KO or NaV1.8-KO Mice

VGLUT2 has also been deleted in TH- or NaV1.8-expressing DRG neurons (here called VGLUT2-TH- or VGLUT2-Nav1.8-KO mice, respectively). VGLUT2-TH-KO mice show reduced sensitivity to nociceptive heat (Table 2), as well as impaired formalin-induced pain responses [6] (Table 2; unfortunately, the effects of peripheral nerve injury in this transgenic mouse have not been analyzed). In VGLUT2-NaV1.8-KO mice, reduced nociceptive mechanical sensitivity (Table 1) and attenuated NGF-induced thermal hyperalgesia (Table 3), but no alterations in nociceptive thermal sensitivity or formalin- and PSNL-evoked pain [27] have been described (Table 2, Table 3 and Table 4).

#### 2.2.5. VGLUT3-DRG-KO Mice

Finally, in the only available study where VGLUT3 is selectively deleted in C-low threshold DRG mechanoreceptors (through the deletion of Runx1; here called VGLUT3-DRG-KO mice) [33], a mild reduction in mechanical (but not thermal) hypersensitivity during carrageenan-induced inflammation was observed, coinciding with data from global homozygote VGLUT3-KO mice [30,31,33]. However, and in contrast with global homozygote VGLUT3-KO mice, no changes in nociceptive or neuropathic pain conditions are described [30,31,33]. This suggests that the effects on pain mechanisms observed in global VGLUT3-KO mice probably involve more than only DRG neurons (e.g., spinal cord, supraspinal areas).

In conclusion, most evidence shows that VGLUT2 and VGLUT3 are the main players in the transmission of various pain modalities, with a base in DRG neurons (although studies on conditional VGLUT1-KO mice to fully exclude its participation in pain mechanisms are still missing). The dramatic impairment in thermal and mechanical hypersensitivities evoked by noxious stimuli, tissue inflammation, or nerve injury in VGLUT2-DRG-KO mice [26], further supported by analysis in mice with more selective deletions [6,27,38,43], clearly show the relevant role of VGLUT2 in pain mechanisms. Interestingly, and particularly during inflammation, concomitant glutamatergic and neuropeptidergic signaling seems necessary for full expression of pain behavior [6,28], in agreement with the peptidergic nature of VGLUT2-expressing DRG neurons [9,29]. The role of VGLUT3 appears to be more selective, mostly participating in the transmission of mechanical and cold hypersensitivities, and being dependent on the type of injury (e.g., no participation during CCI *vs.* participation during SNI-, incisional- and oxaliplatin-induced pain) (see [30,31]).

### 2.3. VGLUT Deletion, Visceral Pain and Itch

As mentioned above, virtually all colorectal and urinary bladder DRG neurons in mice express VGLUT2, and some also VGLUT1 or VGLUT3 (Figure 1), suggesting that changes in their expression should modulate visceral pain. However, global heterozygous VGLUT1- [40] or VGLUT2-KO mice [40] show no differences in their responses to the (rather nonspecific) acetic acid visceral pain test; comparison between global VGLUT3-KO mice and WT mice (same as used in [30]) by analysis of visceromotor responses during noxious colorectal distension also failed to show differences (Brumovsky, unpublished results). Particularly for VGLUT3, the lack of effect could relate to the small number of VGLUT3-expressing DRG neurons innervating the mouse colorectum [8] and urinary bladder [7]. Nevertheless, increases in VGLUT3 expression in DRGs and the prefrontal cortex have been reported in rats suffering visceral hyperalgesia due to intestinal infection with *Trichinella spiralis* [44]. More indirect evidence for a role of VGLUT3 is also available from a study on Runx1-KO mice (same as used in [33]), where impaired mechanical and chemical (serotonin-induced) visceral nociception and reduced colitis-induced mechanical hypersensitivity has been described [45]. However, loss of expression of several receptors associated with nociception, including TRPV1, TRPM8, and others, is also a feature in Runx1-KO mice [46]. Therefore, the contribution of VGLUT3 in DRG neurons to visceral pain is yet to be fully established. Likewise, the role of VGLUT2, the most abundantly expressed transporter in mouse colorectal [8] and urinary bladder [7] DRG neurons (Figure 1A,B), remains to be analyzed, both in transgenic mice and pharmacologically.

Finally, deletion of VGLUT2 exposed an additional unexpected behavior, namely spontaneous itch and resulting skin injuries. Such behavior has been confirmed in VGLUT2-all-DRG- [26], VGLUT2-TRPV1- [6,38,43], and VGLUT2-TH-KO mice [6], and appears to be dependent on concomitant peptidergic signaling [38].

## 3. Mechanisms Associated with VGLUT Deletion and Pain Modulation

In the previous section, we addressed the effects of genetic manipulations of each VGLUT on pain-like behavior in rodents. Global KO strategies result in widespread alterations at different levels of the nervous system, and are therefore difficult to address in terms of the mechanisms involved. In contrast, the mechanisms are easier to discuss in mice with selective deletion of VGLUTs in primary afferent neurons. Deletion of VGLUTs in these neurons most likely alters the release probability of glutamate from their nerve endings, both central and peripheral. In fact, the amount and loading rate of glutamate, the size of the glutamatergic quanta, and the reserve pool of synaptic vesicles are strongly influenced by the number of VGLUT copies [21,41,47,48,49,50]. In support of this conclusion, vesicles with reduced VGLUT expression or with non-functional transporters (point mutations at the pore sites) exhibit lower release probability, as shown in mouse hippocampus synapses in culture [50]. However, an exhaustive analysis of the mechanisms by which VGLUT deletion in primary afferent neurons could affect pain transmission, both in global or conditional KO mice, has yet to be done.

So far, only one study has shown altered quantal release in thalamic neurons in global heterozygous VGLUT2-KO mice that also show, as described above, impaired pain-like behavior [41]. If extrapolated, such data obtained in thalamic neurons leads to the speculation that one basic consequence of VGLUT deletion in primary afferent neurons was the alteration of quantal release at their central projections terminating in the spinal cord. Evidence supporting such an occurrence has been provided in a number of recent studies. On one hand, several receptors associated to the transmission of pain are expressed by VGLUT-expressing primary afferent spinal nerve endings, including TRPV1 [6,25,27,28,38] and the cooling sensor transient receptor potential melastatin 8 (TRPM8; [31])—their activation most certainly results in the induction of glutamate release, which would be compromised in VGLUT-KO mice. On the other hand, selective deletion of VGLUT2 in NaV1.8 DRG neurons (VGLUT2-NaV1.8-KO mice) results in a dramatic reduction in the number of c-Fos immediate early gene (a marker of neurons excited by primary afferent-released excitatory neurotransmitters) in lamina II of the dorsal horn, following peripheral mechanical stimulation of the hindpaw [27]. Likewise, deletion of VGLUT2 from TRPV1-expressing DRG neurons (VGLUT2-TRPV1-KO mice) also results in reduced numbers of c-Fos neurons in laminae I-III of the dorsal horn upon heat stimulation of the hindpaw [26], although this is also influenced by interactions with CGRP and SP neurotransmission [28,38]. Finally, a mechanistic cooperation between VGLUT3-expressing fibers and their TRPM8 activation was shown to induce a stronger facilitation of field potentials in lamina I-II of the dorsal horn [31]. Taken together, it is highly likely that VGLUT2 or VGLUT3 deletion strongly impairs TRPV1- or TRPM8-induced glutamatergic release from primary afferent nerve endings. This would reduce the degree of activation of spinal projection neurons and interneurons, and therefore, alter the transmission of nociceptive signals towards higher levels of the neural axis.

Besides the central effects, VGLUTs may also have a role in the periphery. In mice, VGLUT2 is abundantly represented in primary afferent nerve endings in skin [9] and visceral organs [7,8]. Also, A- and/or C-fiber and capsaicin stimulation of the sciatic nerve, results in increased hindpaw glutamate levels [51], with VGLUT2 as a likely participant during vesicular glutamate loading. Importantly, exogenous application of glutamate in the hindpaw of rats results in C-fiber depolarization and pain-like behavior [51,52,53,54]. Therefore, it is plausible that VGLUT2 deletion and its reduction, also in the peripheral nerve endings of primary afferent neurons, results in reduced glutamate release and activation of glutamatergic receptors in the periphery, as well as impaired pain-like behavior.

Finally, deletion of VGLUTs could also impact the interaction between DRG neurons in an “intraganglionic fashion”. Evidence supporting the existence of somatic intraganglionic release within DRGs has been provided, for example, for neuropeptides [55,56] and ATP [57]. These and other molecules, “on the loose” within the ganglia, could possibly interact with satellite and neighboring neurons [55,56,57]. Interestingly, one feature of VGLUT2 is its association with the plasma membrane in non-visceral [8,9] and visceral [7,8] DRG neurons. It is possible that the higher density of VGLUT2-LI in the plasma membrane of DRG neurons (Figure 1Ae) represents a higher content of glutamatergic synaptic vesicles, ready to participate in somatic release. If such was the case, deletion of VGLUT2 could also impact neuronal communication within DRGs, potentially affecting the degree of excitability derived from different types of painful stimuli. More research is necessary to establish if: (1) in fact, glutamate is released within DRGs; (2) such released glutamate functionally interacts with glial cells and/or neurons; (3) VGLUT deletion does affect (1) and (2).

## 4. Glutamate Biosynthesis and VGLUT Interactions—Possible Implications to Pain

As already mentioned, glutamate biosynthesis and synaptic vesicle loading are essential contributors to glutamatergic neurotransmission. In fact, the cytoplasmic concentration of glutamate directly influences its intravesicular content [58,59]; in other words, the more glutamate is in the cytoplasm, the larger the vesicular content and quanta (amount of released glutamate) [60,61,62]. For example, heterozygous mice with globally deleted GLS (homozygote mice die after birth) show reduced GLS levels and activity [63], as well as impaired mechanical and thermal nociception, and reduced responses during both phases of intraplantar formalin [64]. However, even with an unlimited glutamate supply, lack of VGLUTs dramatically impairs its loading into synaptic vesicles (see above and [21,41,47,48,49,50]). Moreover, the critical association between available cytoplasmic glutamate and its incorporation in synaptic vesicles is reflected by the observation that “the synapse displays a bias for releasing vesicles that are filled with neurotransmitter… and maintains efficiency by favoring the release of vesicles most likely to cause a postsynaptic response” [50].

Nociception and chronic pain may be influenced specifically by the interaction between VGLUTs and GLS. Interestingly, it has been reported that protein expressions of VGLUT2 [65] and GLS [66] are both upregulated in DRG neurons [66] and their projections within the sciatic nerve of rat during CFA-induced arthritis [65,66], all events that would explain the observed increases in peripheral glutamate release [67]. In support of this inference, an increased concentration of excitatory neurotransmitters, including glutamate, has been observed in synovial fluid from patients who underwent diagnostic or therapeutic arthrocenteses due to active arthritis [68,69]; suggesting, as in the rat, increased glutamate synthesis and vesicular concentration, as well as enhanced release probability and quanta. However, while it remains to be established from the protein point of view, CFA-induced hindpaw inflammation in the mouse fails to alter VGLUTs mRNA expression in DRGs [13]. Therefore, more research is needed to better define if and how VGLUTs and GLS interact during peripheral inflammation-induced pain.

One major drawback to analyzing potential interactions between glutamate biosynthesis and vesicular loading during peripheral nerve injury is the lack of information about GLS expression after injury. In contrast, immunohistochemical analysis in lumbar DRGs of mice with sciatic nerve axotomy revealed a reduction in the number of neurons expressing VGLUT1 or VGLUT2, although with a concomitant increase in VGLUT2-like-immunoreactivity (staining intensity) in small neurons [9]. Interestingly, such changes appear to occur at the protein level, since no alterations in the number of VGLUT1 or VGLUT2 mRNA-expressing DRG neurons were observed [13]. A modest downregulation has been detected, however, in the number of VGLUT3 mRNA-expressing DRG neurons after axotomy of the sciatic nerve in mouse [13], although if this is also accompanied by concomitant alterations in protein expression remains unknown. Finally, information is also missing about changes in the expression of protein or transcript for any of the VGLUTs in rat DRGs after peripheral nerve injury [29]. However, the reduction in VGLUT1-like-immunoreactivity in the dorsal and ventral horns of the spinal cord in rat (also seen in mouse [9]) after axotomy of the sciatic nerve suggests that its expression is downregulated in the soma (and therefore also the central projections) of primary afferent neurons [70]. Alternatively, such a decrease could also derive from the degeneration of the central projections of severed primary afferent neurons.

Finally, one hypothesis put forth for the decreased numbers of VGLUT1- and VGLUT2-expressing DRG neurons and the parallel lack of transcriptional changes during peripheral nerve injury in the mouse, is that there is an increased transport of vesicles towards active nerve endings (potentially explaining reduced immunohistochemical detection), which would lead neurons to counteract the higher demand by maintaining transcriptional levels [29]. In such a scenario, an upregulation of GLS after peripheral nerve injury could potentially improve the chances of vesicular glutamate loading. Moreover, and in particular for small (presumably nociceptive) DRG neurons, a parallel upregulation of VGLUT2 protein (see above) after axotomy of the sciatic [9] and pelvic [71] nerves, and potentially also GLS, could result in enhanced spinal glutamatergic nociceptive tone and pain (see [29] for further discussion).

## 5. Conclusions

An increasing body of research suggests that glutamatergic primary afferent neurons together with co-neurotransmitters such as neuropeptides and the type of injury involved define the relative contribution of each VGLUT subtype to nociceptive, inflammatory and neuropathic pain [25,28,31,38]. However, a better insight into the sub-cellular mechanisms involved, for instance, in transgenic mice with compromised VGLUT expression, remains to be provided. Also missing are experiments with pharmacological blockade of VGLUTs (these are currently hampered by the lack of selective VGLUT inhibitors). Finally, it is likely that the mechanisms of glutamate biosynthesis and vesicular filling interact and influence pain transmission. More research will be needed to reveal the exact nature of such interactions.
